# COMET V meeting summary

**DOI:** 10.1186/1745-6215-16-S3-A1

**Published:** 2015-11-24

**Authors:** Sarah L Gorst, Doug G Altman, Jane M Blazeby, Mike Clarke, Elizabeth Gargon, Sean Tunis, Paula R Williamson

**Affiliations:** 1 Department of Biostatistics, University of Liverpool, Liverpool, UK; 2 Centre for Statistics in Medicine, University of Oxford, Oxford, UK; 3 School of Social and Community Medicine, University of Bristol, Bristol, UK and Division of Surgery, Head &amp; Neck, University Hospitals NHS Foundation Trust, Bristol, UK; 4 Northern Ireland Network for Trials Methodology Research, Queen's University Belfast, Belfast, UK; 5 Center for Medical Technology Policy, Baltimore, Maryland, USA

## 

On 20-21^st^ May 2015, more than 150 people with an interest in core outcome sets (COS) gathered at the University of Calgary in Alberta, Canada for the fifth meeting of the COMET Initiative. This was the first annual COMET meeting in North America, and the COMET Management Group are grateful to Cochrane Canada for facilitating the meeting and an excellent joint session on the second day. As well as participants from Canada and the USA, people came from Australia, Brazil, Germany, Portugal and the UK.

Over the next two days, the invited plenary talks were complemented by workshops, posters and contributed presentations. Theresa Radwell (Alberta Cancer Foundation) opened the meeting, welcoming all to Calgary and introducing the importance of engaging patients within research and outcome selection. Paula Williamson (COMET Management Group) then spoke about the COMET Initiative, emphasising that COMET is keen to avoid unnecessary duplication of effort and to facilitate the development of COS. The participants were then introduced to important methodological issues in COS through a series of presentations. John Marshall (St. Michael's Hospital, Toronto) provided a critical care perspective and highlighted that mortality is not always the most important outcome from a patient perspective. Amy Hoang-Kim (University of Toronto) presented a recommendation for a minimal set of core domains for use in distal radius fracture clinical practice and research. Moving on to nephrology, Jonathan Craig and Allison Tong (University of Sydney) overviewed existing standardized outcomes, with preliminary results showing how dialysis free time was the most important outcome to haemodialysis patients.

One of the novel additions in COMET V was a panel discussion showing the importance of COS to different stakeholders. John Fletcher (Canadian Medical Association Journal) described the pros and cons of COS from an editor's perspective. Jordi Pardo (OMERACT) outlined the OMERACT process for developing a COS. Carole Légaré (Health Canada) identified the problems seen by regulators because of inconsistency of safety reporting. John Marshall (Canadian Critical Care Trials Group) spoke about challenging issues faced by the critical care research community. Mike Clarke (COMET) brought all of this together by highlighting the resources that are available through COMET to assist in the development and evaluation of COS. The ensuing discussion highlighted the benefits of COS for journals, how stakeholder involvement and international harmonisation are essential to COS development, the need to consider barriers to uptake of COS for researchers, and recognition of the need for a COS that is in no way restrictive.

The afternoon began with David Moher (Ottawa Hospital Research Institute) speaking about the EQUATOR network, which aims to maximise the value of research by improving conduct and reporting. David highlighted how the evaluation of reporting guidleines and COS is critical. He was followed by a series of presentations which centred around outcomes for paediatric trials. Zafira Bhaloo (University of Alberta) emphasised how the reporting of primary outcomes in pediatric trials is inadequate and encouraged higher standards for reporting and informed selection of outcomes and their measures. Michele Hamm (University of Alberta) discussed how the use of social media to identify patient-centred outcomes in child health did not result in broad reach as a stakeholder engagement strategy. Mufiza Kapadia (The Hospital for Sick Children) ended the session by stressing the importance of involving parents and children in COS development.

Alongside the 17 posters that were available for viewing throughout the first day, four of the people who had submitted abstracts had been selected to give a contributed talk. These began with Carina Benstom (University Hospital RWTH Aachen) who highlighted how the problems caused by inconsistent outcome measures in clinical trials are hardly recognised. Chris Hylton (PaCER) spoke about improvements in the results of patient experiences and outcome analysis, from involving patient and community engagement researchers. Sally Crowe (Crowe Associates Ltd) continued the patient theme, by speaking about how workshops offer context and depth for talking about outcomes. The final contributed talk came from Thomas Kelley (International Consortium for Health Outcomes Measurement) who explained how ICHOM's mission is to define global standard sets of outcome measures that really matter to patients for the most relevant medical conditions. Mike Clarke (COMET Management Group) closed the first day with a presentation about COMET in Canada, which highlighted what Canada can do to increase the use of COS in research, for example by helping to persuade funders that COS should be used in research.

The second day (21^st^ May) was shared with Cochrane Canada and the opening of their Annual Symposium. The opening of the joint session was marked by memories of Dave Sackett and his contribution to evidence based healthcare. Following a minute's silence for the many friends, colleagues and admirers of Dave to remember him, the scientific session began with Kay Dickersin (John Hopkins University) highlighting how groundwork needs to be laid in subject areas where there are a lack of COS and how new methods need to be explored for developing COS. Mike presented findings from a survey of outcomes in Cochrane Reviews, showing the wide variation in outcomes and the lack of COS, at least up to 2013. Holger Schünemann (McMaster University) closed the plenary session by providing an overview of the GRADE evidence to decision frameworks.

Following a break, the participants headed off for one of three COMET workshops. Paula Williamson led the first of these, which focused on the methods for developing what to measure in COS. The workshop introduced methodological issues and considerations involved in developing COS. Workshop 2 was led by Mike Clarke and looked at how COS might be used for randomised trials and Cochrane Reviews. Bridget Young (University of Liverpool) led the third workshop providing an interactive opportunity for the participants to identify the challenges that researchers may encounter when planning to involve patients and carers in COS development.

COMET V allowed a wide variety of stakeholders with an interest in COS development to meet and share experiences, findings, and plans with others. It brought together key scientists and consumers responsible for developing and implementing COS. Patient involvement emerged as a major focus of the meeting with an emphasis on engaging the relevant stakeholders early in the process of COS development. Thoughts were offered for how COMET can evolve both in Canada and the rest of the world. And, challenging questions were posed throughout the meeting, including: How can we ensure that COS are well developed in the first place? Is there a magic number of outcomes to be included in a COS and, if so, what is it? As COMET looks forward to COMET VI, it will seek to meet these challenges, guided by an International Advisory Group, which will include Peter Tugwell (University of Ottawa), one of the founders of OMERACT.

The slides from COMET V presentations can be viewed at: http://www.comet-initiative.org/events/FifthCometMeeting.

